# Impairment of sperm efficiency in mice following short-term nano-titanium dioxide exposure: An experimental study

**DOI:** 10.18502/ijrm.v19i12.10055

**Published:** 2022-01-12

**Authors:** Amirhossein Danafar, Arezoo Khoradmehr, Mahya Hosseini Bondarabadi, Fahime Mazaheri, Amin Tamadon, Soheila Pourmasoumi, Lida Gholizadeh, Mojgan Moshrefi, Iman Halvaei, Akram Hosseini, Jalal Golzadeh, Tahereh Rahiminia, Morteza Anvari

**Affiliations:** ^1^Dr. Seyedhassani Medical Sciences Center, Yazd, Iran.; ^2^The Persian Gulf Marine Biotechnology Research Center, the Persian Gulf Biomedical Sciences Research Institute, Bushehr University of Medical Sciences, Bushehr, Iran.; ^3^Research and Clinical Center of Infertility, Yazd Reproductive Science Institute, Shahid Sadoughi University of Medical Sciences, Yazd, Iran.; ^4^Université Clermont Auvergne, CNRS, LMGE, Clermont-Ferrand, France.; ^5^Medical Nanotechnology and Tissue Engineering Research Centre, Yazd Reproductive Science Institute, Shahid Sadoughi University of Medical Sciences, Yazd, Iran.; ^6^Non-Communicable Diseases Research Center, Rafsanjan University of Medical Sciences, Rafsanjan, Iran.; ^7^Clinical Research Development Unit (CRDU), Moradi Hospital, Rafsanjan University of Medical Sciences, Rafsanjan, Iran.; ^8^Department of Anatomical Sciences, Faculty of Medical Sciences, Tarbiat Modares University, Tehran, Iran.; ^9^Department of Anatomical Sciences, School of Medicine, Isfahan University of Medical Sciences, Isfahan, Iran.; ^10^Stem Cell Biology Research Center, Yazd Reproductive Science Institute, Shahid Sadoughi University of Medical Sciences, Yazd, Iran.; ^11^Gametogenesis Research Center, Fertility and Infertility Center, Kashan University of Medical Sciences, Kashan, Iran.; ^12^Department of Biology and Anatomical Sciences, Shahid Sadoughi University of Medical Sciences, Yazd, Iran.; ^13^Amirhossein Danafar and Arezoo Khoradmehr are both first authors

**Keywords:** Titanium dioxide, Spermatogenesis, Histology, Mouse, Chromatin, TUNEL.

## Abstract

**Background:**

Titanium dioxide nanoparticles (TiO ${}_{2}$ NPs) are widely used in many compounds. Recent evidence has displayed some cytotoxic effects of TiO ${}_{2}$ NPs on male reproduction.

**Objective:**

The effects of TiO ${}_{2}$ NP administration on sperm parameters and chromatin and seminiferous histopathology of male mice were investigated.

**Materials and Methods:**

In this experimental study, 32 NMRI male mice (35 ± 3 gr, 8-12-week-old) were divided into four groups (n = 8/each): treated groups were fed orally with 2.5 (group I), 5 (group II) and 10 (group III) mg/kg/day TiO ${}_{2}$ NPs for 40 days and the control group received phosphate buffered saline. Sperm parameters, DNA integrity and chromatin quality were assessed using chromomycin A3, aniline blue, toluidine blue staining and TUNEL. Hematoxylin eosin staining was performed to measure spermatogenic cells and the total diameter of seminiferous tubules. Also, sex hormone and malondyaldehyde levels were measured.

**Results:**

Abnormal sperm tails rose in group III (28.87 ± 4.91) in comparison with the control group (12.75 ± 3.95). However, chromomycin A3 staining and TUNEL showed higher levels in group III in comparison with the control group, whereas aniline blue and toluidine blue staining showed no differences. A significantly lower spermatogenesis index and lumen parameters were observed in group III. Leydig cell numbers, cellular diameters and the area of the seminiferous tubules were lower in the treated groups. The testosterone level was also lower in these groups and the percentage of malondyaldehyde in the seminal fluid was higher.

**Conclusion:**

Exact mechanisms of TiO ${}_{2}$ NPs are not clear; however, cytotoxic and genotoxic effects of TiO ${}_{2}$ NPs may relate to oxidative stress. Given their widespread use, TiO ${}_{2}$ NPs should be a public health focus of attention.

## 1. Introduction

Titanium dioxide nanoparticles (TiO ${}_{2}$ NPs) have recently become an area of concern due to their use in many compounds from food and medicine to industrial applications (1). Their hydrophilic and photo-catalytic properties make them useful for many purposes. In particular, these two properties are useful for water and air purification (2). Over the past decades, TiO ${}_{2}$ NPs have been used to keep food fresh and as a white additive color for foods and cosmetic products (3, 4). It was previously shown that TiO ${}_{2}$ NPs exhibit low toxicity to humans and animals compared to other nanoparticles (5). However, more recent evidence demonstrates that TiO ${}_{2}$ NPs display some cytotoxic features.

It has been reported that TiO ${}_{2}$ NPs can cross into the testes and that the reproductive system is very sensitive to nanoparticles (6), which may have negative effects on the germ line either by direct effects on germ cells or indirect effects on somatic nursing cells that could lead to infertility (7). It has been shown that TiO ${}_{2}$ NPs can enter mouse Leydig cells and male germ line cells and change gene expression (8). Also, they can cause testicular damage and impaired spermatogenesis in male mice (9).

The quality of sperm parameters is related to spermatogenesis, and oxidative stress, the cytoskeleton and the blood-testis barrier (BTB) have critical roles in the maturation and development of spermatozoa. The BTB can restrict exogenous components from entering into the seminiferous tubules from the blood vessels, and makes a stable environment for germ cells and spermatid growth (10) and cause lower spermatogenesis (9). TiO ${}_{2}$ NPs can downregulate BTB junction-related proteins by activating the mitogen-activated protein kinases (MAPK) signaling pathways, which causes male reproductive dysfunction (10).

The exact mechanism of TiO ${}_{2}$ NP impact on spermatozoa dysfunction is still unknown. However, it is proposed that nanozied particles such as TiO ${}_{2}$ NPs can increase reactive oxygen species (ROS) in cells (11). ROS play a critical role in the function of sperm and germ line cells, which can reduce male fertility (12). Normal levels of ROS in seminiferous tubules can have detrimental effects on the fertilization rate by affecting sperm functions including sperm maturation, hyperactivation, capacitation and the acrosome reaction. Generation of ROS causes oxidative damage in molecular structures. High levels of ROS can damage the plasma membrane due to lipid peroxidation, protein denaturation and DNA damage (13).

Some studies suggest that TiO ${}_{2}$ NPs may disrupt endocrine functions and the regulation of the sex hormone profile (14, 15). It has been reported that TiO ${}_{2}$ NPs can induce accumulation of mutations and lead to genotoxicity in somatic cells. Intraperitoneal administration of TiO ${}_{2}$ NPs can significantly impact the reproductive system and increase sperm abnormalities (16).

Given the wide use of TiO ${}_{2}$ NPs, the purpose of this study was to examine the impact of TiO ${}_{2}$ NPs on spermatogenesis, as well as the histomorphometric cytotoxic effects of TiO ${}_{2}$ NPs in male mice. This study presents new data about sperm DNA integrity, sperm morphology and seminiferous tubule histopathology after daily oral TiO ${}_{2}$ NP administration.

## 2. Materials and Methods

### Animal housing and treatment

In this experimental study, 8-12-wk-old NMRI male mice (35 ± 3 gr) were maintained in standard cages and animal house conditions (normal pellets and water ad libitum, humidity 60 ± 10%, room temperature 23 ± 2°C, and 12 hr light/dark cycle) in Yazd Reproductive Sciences Institute, Yazd, Iran. TiO ${}_{2}$ NPs (anatase/rutile, 99+%, 20 nm, Nanosony Co, Iran) were dissolved in water and the suspension was homogenized with a sonicator for 10 min. The TiO ${}_{2}$ NP suspension was prepared at the concentrations of 2.5, 5 and 10 mg/kg just before use in water (9). The mice were divided into four groups (n = 8/each group), including a control group and three treated groups (17). The control group received water intragastrically. The group I, group II and group II treated groups were fed orally with 2.5, 5 and 10 mg/kg/day TiO ${}_{2}$ NPs for 40 days, respectively (9).

**Figure 1:**
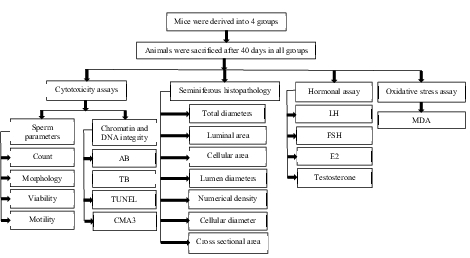
The four groups were sacrificed after 40 days, and cytotoxicity, hormonal and oxidative stress assays and seminiferous tubule histopathology were evaluated in all treated groups in comparison with the control group. AB: Aniline blue, CMA3: Chromomycin A3, E2: Estradiol, FSH: Follicle stimulating hormone, LH: Luteinizing hormone, MDA: Malondyaldehyde, PAP: Papanicolaou, TB: Toluidine blue, TUNEL: Terminal deoxynucleotidyl transferase dUTP nick end labeling.

After the 40 days (which is longer than needed for spermatogenesis in mice), the mice were sacrificed by cervical dislocation and the caudal epididymis was transferred into a petri dish. The testis was separated and fixed in Bouin's solution for histological assessment. The suspension containing caudal epididymis and 1000 µl of Ham's F10 medium (Vitrolife, Gothenburg, Sweden) was incubated for 30 min at 37°C in a humidified atmosphere of 5% CO ${}_{2}$ (17). After 30 min, sperm parameters were analyzed, and sperm DNA integrity and chromatin quality were assessed using standard procedures. All assays were summarized in figure 1.

### Sperm parameters assessment

Sperm count was assessed by Makler's counting chamber. To assess sperm motility, the percentages of sperm motility were classified into three classes of progressive, non-progressive and immotile sperm. Papanicolaou (Merck, Germany) and eosin-nigrosin staining (Merck, Germany) were used to examine sperm morphology and viability, respectively (18). In all groups, sperm viability was evaluated 30 min after sperm motility assessment. Sperm morphological abnormalities were classified as head (size, shape, double head, acrosomal section), neck (bent, cytoplasmic droplets) and tail (bent, coiled, double tail, cytoplasmic droplets). Eosin-nigrosin staining was used to assess sperm viability; white (unstained) and pink (or red) sperm heads were classified as live and dead sperm, respectively. To evaluate sperm motility, morphology and viability, 200 sperm were evaluated under a light microscope at x400 magnification (Motic, Spain).

### Assessment of sperm DNA integrity and chromatin packaging quality

#### Terminal deoxynucleotidyl transferase dUTP nick end labeling (TUNEL) assay

For detection of sperm DNA fragmentation, the TUNEL assay was applied. The slides were ﬁxed with 4% paraformaldehyde, washed for 1 hr in phosphate-buffered saline, and then treated with 3% H ${}_{2}$ O ${}_{2}$ and 0.1% Triton X-100 for 15 and 20 min, respectively. Afterward, the slides were incubated with enzyme (Roche, Germany): label solution (5:45 µl) for 1 hr. For positive and negative controls, the slides were incubated with DNase I grade I (3 U/ml in 50 mM Tris-HCl, pH 7.5, 1 mg/ml BSA) and 50 µl of label solution (without terminal transferase), respectively. Finally, for each slide, 200 sperm were evaluated under a fluorescence microscope at x400 magnification (Olympus BX51, Japan) (17) and were counted.

#### Chromomycin A3 staining

Protamine deficiency in sperm chromatin can be detected using chromomycin A3 assay. Methanol: glacial acetic acid (3:1) was used to fix the air-dried slides in a refrigerator. The fixed slides were treated with 100 µl of chromomycin A3 solution (Sigma, Germany) for 10 min and then, the slides were washed with McIlvain's buffer. For each slide, 200 sperm were evaluated under a fluorescent microscope at x400 magnification (Olympus BX51, Japan) with a 460-470 nm filter (17).

#### Toluidine blue (TB) staining

TB staining was used to determine chromatin condensation and DNA integrity via binding to phosphate groups of DNA strands. Ethanol: acetone (1:1) was used to fix the slides at 4°C for 30 min; these were then hydrolyzed in 0.1N HCl for 5 min. Then, the slides were rinsed three times in distilled water and finally, the samples were stained in 5% TB (Sigma, Germany) in 50% McIlvain's citrate phosphate buffer (pH: 3.5) (17). For each slide, 200 sperm were evaluated under a light microscope at x400 magnification (Olympus BX51, Japan).

#### Aniline blue (AB) staining

The acidic staining of AB reacts with lysine residues in histone proteins and leads to detection of sperm chromatin remodeling (19). 3% buffered glutaraldehyde in 0.2 M phosphate buffer (pH: 7.2) was used to fix the air-dried smears for 30 min. Then, the slides were stained with 5% AB (Sigma, Germany) in 4% acetic acid (pH: 3.5) for 5 min. Finally, the slides were washed in distilled water and 200 sperm were evaluated under a light microscope at x400 magnification (Olympus BX51, Japan).

### Histomorphometric evaluation and cell numbers of seminiferous tubules

The five testis samples were fixed in Bouin's solution for each group. Tissues were dehydrated in a graded alcohol series, cleared in xylene and embedded in paraffin wax. Samples were cut in 7 m (five slices from each testis) and then glass slides were stained with hematoxylin and eosin. A histopathologist unaware of the treatments evaluated the histological slides after hematoxylin-eosin staining. Total diameters, lumen diameter and cellular diameter (µm), luminal area, cellular area and cross-sectional area ( × 10^4^ μm^2^) and numerical density (20) were measured using stereological methods under an optical microscope at x100 magnification (Olympus BX51, Japan). The mean seminiferous tubule diameter, and cross-sectional and numerical density area were calculated (21). For each slide, the mean of 10 random fields was calculated for measurements of Sertoli, Leydig, spermatogonia, primary spermatocyte and spermatid cell numbers (22) at x400 magnification. For the TUNEL assay in the testes for detection of cell apoptosis, sections were dewaxed in xylene and rehydrated through a graded series of ethanol, then washed for 1 hr in phosphate-buffered saline. The assay was performed according to the manufacturer's instructions; sperm DNA fragmentation was detected under a fluorescence microscope (Olympus BX51, Japan) (23).

### Plasma sex hormone levels

Serum samples were evaluated for their content of sex hormones (luteinizing hormone, follicle stimulating hormone, estradiol (E2) and testosterone), using the radioimmunoassay technique (DIA source Immuno-assays, S.A., Belgium), according to the manufacturer's instructions (20).

### Malondyaldehyde (MDA) assay

Seminal MDA levels were measured using a method that was based on a thiobarbituric acid (TBA) reaction and extraction with normal butanol (24), and 31.35 mg MDA (1 mM) and 57.66 mg TBA (4.0 mM) separately were dissolved in 100 mL glacial acetic acid. Semen was mixed with MDA and TBA solutions (1:1). After that, the seminal mixture was boiled in a water bath at 95°C for 60 min. The mixture was cooled at room temperature and spectrophotometric detection of absorbance was done at 532 nm wavelength and compared with a standard curve (25).

### Ethical considerations

Animal treatment methods were approved by the Ethics Committee of Yazd Reproduction Sciences Institute, Shahid Sadoughi University of Medical Sciences, Yazd, Iran (Code: IR-SSU-RSI-REC-2169).

### Statistical analysis

The Shapiro-Wilk test was applied to assess the normal distribution of the data. One-way ANOVA followed by Tukey post hoc test were used for comparison of the data between the different groups using the Statistical Package for the Social Sciences (SPSS), version 20 (SPSS Inc., USA). The data were presented as mean ± standard deviation. Analyses were considered as statistically significant at p < 0.05.

## 3. Results

### Sperm parameter assessment

**Figure 2:**
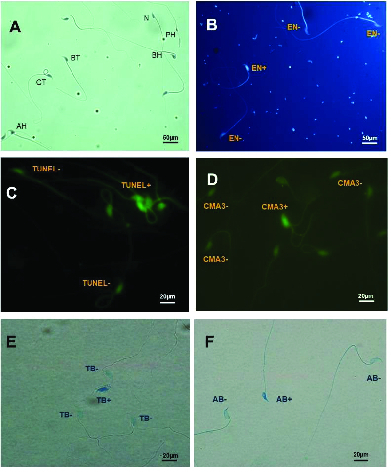
A) Sperm morphology determined using Papanicolaou staining: N, Normal sperm; PH, Pin head with residual body in neck, BH, Bent head, CT, Coiled tail; BT, Bent tail, AH, Amorphous head. B) Eosin-nigrosin staining: live sperms are white (unstained) sperm heads (EN+) and dead sperms are pink (or red) (EN-) C) TUNEL assay: Positive apoptosis cells are brilliant florescent green (TUNEL+) and negative apoptosis cells are pale and opaque green (TUNEL-). D) CAM3 Staining: Positive and negative CAM3 were considered luminous yellowish and without brightness perms, respectively. E) Toluidine blue (TB): Sperm cells with normal chromatin are light blue (TB-) and abnormal chromatin is dark blue (TB+). F) Aniline blue (AB) staining: Sperm head with immature and mature nuclear chromatin are intense dark blue (AB+) and light blue (AB-), respectively ( × 400 magnification).

As shown in table I, the sperm count was lower in group III when compared to the control group, but this was not statistically significant (p = 0.49). It was found that the percentage of progressive motility was significantly higher in the control group than in groups II and III p ≤ 0.001 for both). The percentage of progressive motility was approximately 14% and 15% lower in the groups that received 5 and 10 mg/kg, respectively, compared to the control group. It was clear that there were no significant effects of different concentrations of TiO ${}_{2}$ NPs on the non-progressive sperm in the treated groups compared to the controls (p = 0.07). Moreover, the treatment of mice with different dosages of TiO ${}_{2}$ NPs was associated with a higher percentage of immotile sperm in groups II and III, compared to the control group (p ≤ 0.001). Normal morphology (Figure 2A) was significantly lower in all treated groups compared to the control group (Table I). In addition, sperm viability (Figure 2B) in groups II and III was approximately 16% and 22% less than in the control group (Table I).

### Sperm DNA integrity and chromatin packaging quality

In the TUNEL assay, the apoptotic sperm cells exhibited intensive and brilliant fluorescent green, while the natural cells displayed pale and opaque green (Figure 2C). As shown in table II, a slight difference was observed in groups II and III compared to the control group in the TUNEL assay (p = 0.04) (Figure 2D). The results of CMA3 staining showed higher levels of abnormal sperm in group III in comparison with the control group (p = 0.05). In TB staining, sperm with normal chromatin were seen as colorless but sperm with chromatin abnormalities were observed as dark blue (Figure 2E). In AB staining, sperm with a dark blue and unstained nucleus were considered as having abnormal and normal chromatin, respectively (Figure 2F). The rate of abnormal chromatin in the AB and TB staining was higher in all treated groups compared with the control group, but this difference was not significant (p = 0.08 and p = 0.09, respectively) (Table II).

### Stereological analysis

**Figure 3:**
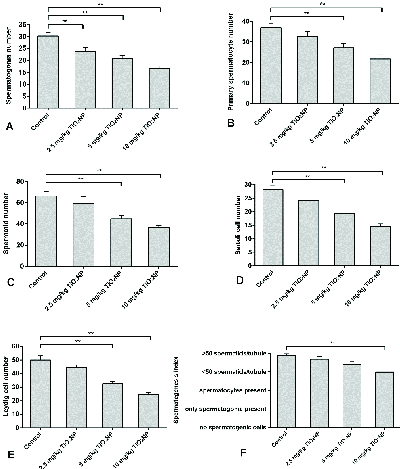
Mean and standard error of cell number in seminiferous tubules in 2.5, 5 and 10 mg/kg groups in comparison with controls after 40 days of TiO ${}_{2}$ NP exposure. A) Spermatogonia cell number, B) Primary spermatocyte cell number, C) Spermatid cell, D) Sertoli cell number, E) Leydig cell number, F) Spermatogenesis index. *P < 0.05, **P < 0.01.

TiO ${}_{2}$ NP exposure was associated with differences in spermatogonia numbers compared to the control group (p < 0.01). Significantly fewer spermatogonia were found in groups I, II and III compared to the control group. Groups II and III displayed fewer primary spermatocytes and spermatid cells compared to controls 40 days after TiO ${}_{2}$ NP exposure (p = 0.02). There were significantly fewer Sertoli and Leydig cells in groups II and III (p ≤ 0.001). Overall, a significantly lower spermatogenesis index (p = 0.03) was observed in group III, but not in groups I and II, in comparison with the control group. The results of the histopathology assessments are shown in figure 3.

**Figure 4:**
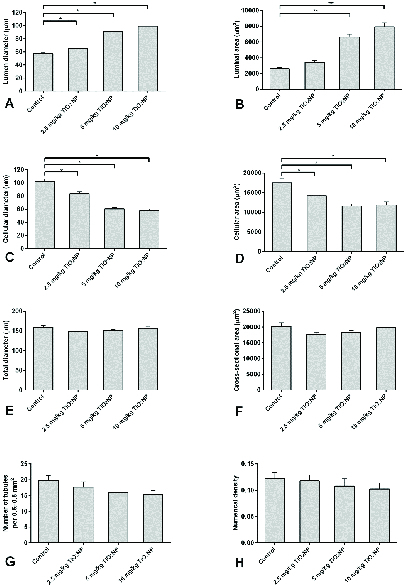
Mean and standard error of stereological indices of seminiferous tubules in 2.5, 5 and 10 mg/kg groups in comparison with controls after 40 days of TiO ${}_{2}$ NP exposure. A) Lumen diameter (μm), B) Luminal area (μm^2^), C) Cellular diameter (μm), D) Cellular area (μm^2^), E) Total diameters (μm), F) Cross-sectional area of the tubule (μm^2^), G) Number of seminiferous tubules per unit area of testis, H) Numerical density of the seminiferous tubules. *P < 0.05, **P < 0.01.

**Figure 5:**
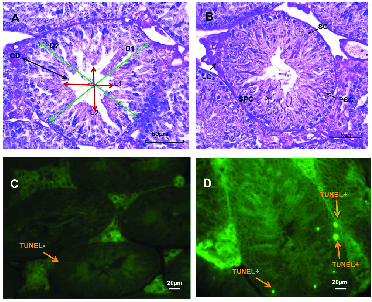
A) Seminiferous tubules in normal testes. D1 and 2: Total diameter (Green arrows), L1 and 2: Lumen diameter (Red arrows), CD: Cellular diameter (Black arrow) (Magnification, hematoxylin-eosin staining). B) Spermatogenesis in seminiferous tubules. LC: Leydig cell, SC: Spermatogonia cell, SPC: Spermatid cell, PSC: Primary spermatocyte cell. C) Normal and D) Apoptotic cells in seminiferous tubules using TUNEL assay, ( × 400 magnification).

In groups I, II and III, the lumen diameter was significantly longer compared to in the control animals (p ≤ 0.001) (Figure 4A), and also there was a significantly larger lumen area in groups II and III (Figure 4B). The cellular diameters and area of the seminiferous tubules were smaller in groups I, II and III (Figures 4C and D). The total diameter and cross-sectional area were not varied across the groups (Figures 4E and F). However, there was a smaller number of tubules and density in all exposed groups compared to the control group, though this was not significant (Figures 4G and H). The results of the lumen studies are shown in figures 4 and 5. Also, there were significantly more apoptotic cells in the seminiferous tubules of groups II and III compared to in the control group (p = 0.02) (Table II).

### Sex hormone levels and MDA 

The effects of TiO ${}_{2}$ NPs on serum hormones of male mice are shown in table III. Following TiO ${}_{2}$ NP exposure, luteinizing hormone, follicle stimulating hormone and E2 levels were not different in the groups, whereas testosterone was markedly lower in group III compared with the control animals (p ≤ 0.001). The percentage of MDA in the group III seminal fluid was higher when compared with the control group (Table III).

**Table 1:** Comparison of sperm parameters in the control, 2.5 (group I), 5 (group II) and 10 (group III) mg/kg groups


	**Group**				
**Sperm parameters**	**Control**	**Group I**	**Group II**	**Group III**	**p-value**
**Count ( × 10^6^/ml)**	28.38 ± 6.54	27.37 ± 6.18	26.25 ± 7.75	25.00 ± 6.34	0.49
**Progressive motility (%)**	52.75 ± 6.60	49.87 ± 14.09	41.88 ± 9.37 ${}^{**}$	37.75 ± 10.09 ${}^{**}$	< 0.01
**Non-progressive motility (%)**	19.62 ± 3.24	18.98 ± 8.78	20.50 ± 8.56	21.50 ± 6.21	0.07
**Immotile sperm (%)**	27.63 ± 6.98	31.15 ± 7.96	37.62 ± 11.78 ${}^{*}$	40.75 ± 9.88 ${}^{*}$	< 0.01
**Normal morphology (%)**	83.00 ± 5.90	62.50 ± 9.54 ${}^{**}$	57.12 ± 6.05 ${}^{**}$	51.87 ± 19.29 ${}^{**}$	< 0.01
**Viability (%)**	72.87 ± 5.84	69.00 ± 6.90	56.00 ± 9.90 ${}^{*}$	50.37 ± 13.01 ${}^{**}$	< 0.01
Values presented as Mean ± SD. One-way ANOVA followed by a Tukey post hoc test were used for comparison of the data between the different groups. *P < 0.05, **P < 0.001 when compared to the control group					

**Table 2:** Cytochemical tests for sperm chromatin quality and DNA fragmentation in the control, 2.5 (group I), 5 (group II) and 10 (group III) mg/kg groups


	**Group**				
**Sperm assays**	**Control**	**Group I**	**Group II**	**Group III**	**p-value**
**TUNEL in sperm (%)**	5.50 ± 2.17	6.00 ± 3.20	10.00 ± 9.2*	11.50 ± 2.17*	0.04
**CMA3 (%)**	21.00 ± 2.34	23.00 ± 6.9	24.32 ± 6.9	26.43 ± 5.11*	0.05
**AB (%)**	13.49 ± 4.9	15.00 ± 4.00	14.42 ± 4.40	16.39 ± 2.8	1.94
**TB (%)**	11.00 ± 6.00	12.94 ± 0.01	13.00 ± 3.5	14.76 ± 0.40	1.28
**TUNEL in seminiferous tubules (%)**	8.32 ± 5.12	9.05 ± 8.42	15.85 ± 3.8*	17.16 ± 7.71*	0.02
Values presented as Mean ± SD. One-way ANOVA followed by a Tukey post hoc test. *P < 0.05 when compared to the control group. TUNEL: Terminal deoxynucleotidyl transferase mediated d-UTP nick end labelling assay, CMA3: Chromomycin A3, AB: Aniline blue, TB: Toluidine blue					

**Table 3:** Comparison of serum hormones and MDA in the control, 2.5 (group I), 5 (group II) and 10 (group III) mg/kg groups


	**Group**				
**Parameters**	**Control**	**Group I**	**Group II**	**Group III**	**p-value**
**LH (AU/ml)**	1.86 ± 0.23	1.72 ± 0.44	1.67 ± 0.52	1.66 ± 0.53	0.99
**FSH (AU/ml)**	0.96 ± 0.04	0.91 ± 0.03	0.90 ± 0.03	0.89 ± 0.02	0.59
**E2 (AU/ml)**	0.90 ± 0.25	0.89 ± 0.22	0.87 ± 0.25	0.82 ± 0.19	0.12
**Testosterone (AU/ml)**	5.08 ± 1.68	3.99 ± 1.86	3.97 ± 1.51	1.99 ± 1.28*	0.01
**MDA (µM)**	0.010 ± 0.001	0.009 ± 0.001	0.008 ± 0.003	0.02 ± 0.003**	≤ 0.001
Values presented as Mean ± SD. One-way ANOVA followed by a Tukey post hoc test were used for comparison of the data between the different groups. *P < 0.05, **P < 0.001 when compared to the control group. LH: Luteinizing hormone, FSH: Follicle stimulating hormone, E2: Estradiol, MDA: Malondyaldehyde assay					

## 4. Discussion

It has been suggested that industrial NPs may increase the risk and incidence of reproductive disorders, and that occupational and environmental exposure to TiO ${}_{2}$ NPs may be one of the risk factors for increased infertility in humans (6). The present results showed that the most abnormal morphology was observed in sperm tails and the percentage of abnormal heads and necks was approximately six-fold higher in group III than in the control group. There were significantly more apoptotic sperm and the rate of abnormal chromatin in CMA3, AB and TB staining was significantly higher in the groups exposed to TiO ${}_{2}$ NPs, compared with the controls. A significantly lower spermatogenesis index, lumen parameters and testosterone level were observed and the percentage of MDA in seminal fluid was higher as well.

In 2020, a study reported that the administration of TiO ${}_{2}$ NPs led to negative effects on sperm parameters including count, motility and morphology (26). This was similar to the results of the present study. In another study, data showed that exposure to TiO2NPs and zinc oxide nanoparticles was associated with decreased sperm number and motility and with increased abnormal sperm in experimental mice (16). They concluded that TiO ${}_{2}$ NPs may pass through the BTB and create harmful damage to testes and sperm.

Small amounts of ROS are necessary for normal sperm functions such as capacitation and the acrosome reaction. High ROS levels can reduce normal sperm morphology and motility by peroxidation of spermatozoa mitochondria and flagellum structure. It has been shown that TiO ${}_{2}$ NPs produce ROS in animal models (13). It reported that high levels of ROS in seminal fluid have a positive correlation with abnormal sperm morphology (27). Our findings were similar to another study which demonstrated that sperm parameters (count, motility, viability and morphology) were reduced in the epididymis tail after frequent consumption and high dosage of TiO ${}_{2}$ NPs in male mice (28).

In the present study, MDA in seminal fluid and sperm viability were significantly lower in group III. We suggest that TiO ${}_{2}$ NPs may induce ROS generation in the testis, and that high levels of ROS are responsible for the increase in abnormal sperm morphology and reduced sperm viability. Another study showed that sperm ﬂagellum abnormalities were significantly more common in the treated groups vs. the controls after 1, 2 or 5 days (29). To the best of our knowledge, this is the first study to analyze abnormal sperm morphology characteristics separately in three classifications (head, neck and tail defects) after TiO ${}_{2}$ NP exposure.

The BTB provides a unique environment for spermatogenesis and to control the transportation of components from vessels to the seminiferous tubules. It has been found that TiO ${}_{2}$ NPs activate the MAPK signal pathways, which decreases BTB junction-related proteins and finally destroys the normal BTB structure. Furthermore, MAPK signal pathways interfere with some cellular processes such as cell proliferation, cell differentiation, and apoptosis. It has been reported that activation of MAPK signal pathways results in related oxidative stress as well (28). It is known that TiO ${}_{2}$ NPs can induce apoptosis in testicular germ cells and epididymal sperm in laboratory animals (30, 31). This is the first study to examine the effects of TiO ${}_{2}$ NPs on mouse sperm DNA fragmentation by TUNEL assay and chromatin condensation tests including CMA3, AB, and TB. It has previously been shown that sperm chromatin defects are associated with apoptosis (32). Our results showed that the rate of AB positive sperm in all treated groups was higher than in the control group, but this was not significant. Consequently, it can be concluded that TiO ${}_{2}$ NPs probably do not substantially affect the histone profile of sperm chromatin when exposed to a concentration of 10 mg/kg for a short time, and a higher concentration may be needed. It can be hypothesized that the lower quality of sperm chromatin observed was probably caused by the toxic effects of TiO ${}_{2}$ NPs and hormonal profile defects.

It reported that exposure to TiO ${}_{2}$ NPs in different doses for 60 sequential days in male animals can cause many defects in testicular tissue, Sertoli cells and spermatogenic cell lines including elevation in apoptotic cells and variation in seminiferous tubule diameter (33). Apoptosis has an important role during spermiogenesis and proliferation of germ cells with the surrounding Sertoli cells (34). In one study, apoptotic cells in seminiferous tubules and interstitial spaces increased, and interstitial spaces revealed enlarged testicular histology after intraperitoneal injection of 2.5 and 5 mg/kg over 1, 2, 3, and 5 wk (29). In another, in the seminiferous tubules, the spermatogenic cell layers reduced in the groups with 50 and 250 mg/kg of body weight compared to the control group, but no histological changes were detected (35).

It has been reported that 30-80% of male infertility is related to oxidative stress (12). In mice, oxidative stress after TiO ${}_{2}$ NP exposure induces peroxidation of protein and DNA, leading to reduced Leydig cells and testosterone levels, which results in reduced spermatogenesis. This is because testosterone, released by the Leydig cells, is an essential factor for the spermatogenesis process (36). Testosterone and E2 are necessary for normal reproductive function. TiO ${}_{2}$ NPs alter feedback regulation of estrogen synthesis, which reduces testosterone and E2. It has been suggested that alteration in serum testosterone during TiO ${}_{2}$ NP exposure leads to reduced spermatogenesis and has a pivotal role in activating apoptosis in the spermatogonial cells (28).

## 5. Conclusion

Overexpression of the MAPK signal pathway after TiO ${}_{2}$ NP exposure disrupts the structure and function of the BTB, which may damage spermatogenesis. In this study, TiO ${}_{2}$ NP exposure likely increased oxidative stress, affecting testicular function by activating the MAPK signaling pathway and disrupting the BTB, leading to male reproductive dysfunction. Exact TiO ${}_{2}$ NPs mechanisms are not clear; however, they may relate to oxidative stress. According to our data, we can conclude that sperm function may be disrupted through cytotoxic and genotoxic effects of TiO ${}_{2}$ NP administration. Further investigation is required to reveal the underlying mechanisms. Considering the widespread use of TiO ${}_{2}$ NPs and the reported detrimental effects of them, this public health issue should be the focus of attention.

##  Conflict of Interest

The authors have no financial or nonfinancial conflict of interest.
